# Efficacy of a live attenuated vaccine in classical swine fever virus postnatally persistently infected pigs

**DOI:** 10.1186/s13567-015-0209-9

**Published:** 2015-07-09

**Authors:** Sara Muñoz-González, Marta Perez-Simó, Marta Muñoz, José Alejandro Bohorquez, Rosa Rosell, Artur Summerfield, Mariano Domingo, Nicolas Ruggli, Llilianne Ganges

**Affiliations:** Centre de Recerca en Sanitat Animal (CReSA)-IRTA, Campus de la Universitat Autònoma de Barcelona, 08193 Bellaterra, Barcelona, Spain; Departament d’Agricultura, Ramaderia, Pesca, Alimentació i Medi Natural, (DAAM), Generalitat de Catalunya, Catalunya, Spain; Institute of Virology and immunology (IVI), Mittelhäusern, Switzerland; Departament de Sanitat i d’Anatomia Animals, Facultat de Veterinària, UAB, 08193 Bellaterra, Barcelona, Spain

## Abstract

Classical swine fever (CSF) causes major losses in pig farming, with various degrees of disease severity. Efficient live attenuated vaccines against classical swine fever virus (CSFV) are used routinely in endemic countries. However, despite intensive vaccination programs in these areas for more than 20 years, CSF has not been eradicated. Molecular epidemiology studies in these regions suggests that the virus circulating in the field has evolved under the positive selection pressure exerted by the immune response to the vaccine, leading to new attenuated viral variants. Recent work by our group demonstrated that a high proportion of persistently infected piglets can be generated by early postnatal infection with low and moderately virulent CSFV strains. Here, we studied the immune response to a hog cholera lapinised virus vaccine (HCLV), C-strain, in six-week-old persistently infected pigs following post-natal infection. CSFV-negative pigs were vaccinated as controls. The humoral and interferon gamma responses as well as the CSFV RNA loads were monitored for 21 days post-vaccination. No vaccine viral RNA was detected in the serum samples and tonsils from CSFV postnatally persistently infected pigs for 21 days post-vaccination. Furthermore, no E2-specific antibody response or neutralising antibody titres were shown in CSFV persistently infected vaccinated animals. Likewise, no of IFN-gamma producing cell response against CSFV or PHA was observed. To our knowledge, this is the first report demonstrating the absence of a response to vaccination in CSFV persistently infected pigs.

## Introduction

Classical swine fever (CSF) is one of the most devastating diseases for the pig industry throughout the world affecting both domestic pigs and wild boars [1,2]. It is endemic in Asia, areas of Central and South America and in many Eastern European countries [3,4] with sporadic occurrence in Western Europe. The CSF virus (CSFV), the etiological agent of CSF, is an icosahedral and enveloped positive stranded RNA virus that, together with bovine viral diarrhoea virus (BVDV) and border diseases virus, belongs to the *Pestivirus* genus of the *Flaviviridae* family [5].

As with many other diseases affecting livestock, the most efficient vaccines currently available against CSFV are live attenuated and were developed over 50 years ago [4,6]. The HCLV vaccine was developed in China, by passage in rabbits. Because of its high efficacy and safety, the HCLV vaccine was introduced into many other countries and became known as the Chinese vaccine strain (C-strain) [7]. Immune responses elicited by these vaccines do not allow differentiating infected from vaccinated animals (DIVA). However, live attenuated vaccines are still used in endemic countries. Furthermore, although intensive control programs have been implemented for over 20 years, the virus is still circulating in these regions; therefore, the disease has not been eradicated. Various degrees of CSF severity can be observed, ranging from acute to chronic or subclinical forms.

Recent molecular epidemiology studies from some endemic countries suggest that the virus circulating in the field has evolved under the positive selection pressure exerted by the immune response to the vaccine, leading to new attenuated viral variants that reproduce milder forms of CSF disease [3,8]. On the other hand, moderate virulence strains were found throughout Europe, as in the case of the Catalonia strain responsible for the 2001–2002 CSFV outbreak in Spain that caused mild and nonspecific clinical signs of CSF, an outbreak that was controlled using a non-vaccination policy by stamping-out strategy [9,10].

Recent work by our group demonstrated that persistently infected piglets can be generated by early postnatal infection with CSFV of low and moderate virulence [11]. For six weeks after postnatal infection, most of the piglets remained clinically healthy, despite persistent high virus titres in the serum, tissues, nasal and rectal swabs. Notably, these animals were unable to mount any detectable humoral and cellular immune response. At necropsy, the most prominent gross pathological lesion was severe thymus atrophy. Contrary to persistent infection, animals developing the chronic form of CSF are able to generate a specific immune response against the virus, mainly an antibody response [4,12,13].

Considering the CSF epidemiological situation in endemic areas, where low virulence strains are prevalent [3,4,8,14,15] and the epidemiological implications that persistently infected animals can exert in the eradication of the disease (revised in [16,17]) we studied the immune response to a live attenuated CSFV vaccine in six-week-old CSFV postnatally persistently infected pigs. Interestingly, none of the vaccinated persistently infected piglets developed a detectable immune response after vaccination. In addition, a complete lack of viral RNA was detected in the serum samples and tonsils from CSFV postnatally persistently infected pigs during the 21 days post-vaccination (dpv). These results have important implications for vaccine control programs in the endemic context.

## Materials and methods

### Cells and viruses

PK-15 cells (ATCC CCL 33) were cultured in DMEM medium, supplemented with 10% pestivirus-free foetal bovine serum (FBS) at 37 °C in 5% CO2. The cells were infected with 0.1 TCID_50_/cell in 2% FBS, and the virus was harvested 48 h later. Peroxidase-linked assay (PLA) [18] was used for viral titration following the statistical methods described by Reed and Muench [19]. The Catalonia 01 strain belongs to the CSFV 2.3 genogroup [3], was isolated from CSF Spanish epizootic in 2000–2001 [9,10] and was the strain that originated the persistently infected pigs used in this study [11]. The HCLV vaccine (C-strain) belongs to CSFV 1.1 genogroup and was used in Spain in the 1980s for CSF control. This vaccine has 100% homology with the Z46258 strain into the N^pro^ region [7]. Finally, the Thiverval vaccine strain (provided by Pasteur Institute, Romania) was used as the stimulus in the Elispot assay for detecting CSFV-specific interferon-gamma (IFN-γ) producing cells. This strain belongs to the CSFV 1.1 genogroup [20].

### Experimental design

To elucidate the immune response induced by the HCLV vaccine (C-strain) in postnatally CSFV persistently infected pigs, two groups with four domestic pigs each at six weeks old were vaccinated with a pig dose (equivalent with 100 Protective Doses (PD) by intramuscular injection in the neck. Group 1 included four CSFV postnatally persistently infected pigs born in a biosafety level 3 (BSL3) animal facility (CReSA, Barcelona, Spain) [11], numbered from 1 to 4. These pigs, which had been intranasally infected in the first 8 h after birth with the CSFV Catalonia 01 strain, were viraemic and apparently healthy at six weeks old (study time), although they lacked a humoral response [11].

The second group (Group 2), housed in an independent isolation unit at the BSL-3 facility of CReSA, consisted of four pigs (numbered 5–8) from a sow of the same origin as Group 1. Group 2 was free from *Pestivirus*, porcine circovirus type 2 and porcine reproductive respiratory syndrome virus. Both groups had an average weight of 12.6 kg per pig.

Serum, whole blood samples, nasal and rectal swabs were taken at 0, 4, 8, 13, 15 and 21 dpv. The tonsils were collected at the time of necropsy (21 dpv). The experiments were approved by the Ethics Committee for Animal Experiments of the Autonomous University of Barcelona (UAB) according to existing national and European regulations.

### Clinical signs evaluation after vaccination

A trained veterinarian recorded rectal temperature and clinical signs daily in a blinded manner. The pigs were scored daily as follows: one point: pyrexia; two points: pyrexia + mild clinical signs; three points: severe clinical signs; and four points: death. After euthanasia (with intravenous pentobarbital sodium injection), animals were subjected to an exhaustive necropsy in which pathological signs in different organs and tissues were evaluated.

### PBMCs collection and performing the ELISPOT assay for the detection of CSFV-specific IFN-γ producing cells

Blood collected in 5 mM EDTA at 15 dpv was used to obtain peripheral blood mononuclear cells (PBMCs) by density-gradient centrifugation with Histopaque 1077 (Sigma). The total number of recovered live PBMCs was obtained by staining with trypan blue [21]. The Elispot assay to detect CSFV-specific IFN-γ cells was performed as previously described by Tarradas et al. [22]. Briefly, 5 × 10^5^ live PBMC/well were plated in duplicate at 0.1 multiplicity of infection (MOI) of CSFV Catalonia or Thiverval strain at 0.01 MOI. As controls, duplicate of cells were incubated in the presence of mock-stimulated wells and Phytohaemagglutinin (PHA) (10 μg/mL). The counts of spots in the media for mock-stimulated wells were considered as the baseline for the calculation of antigen-specific frequencies of IFN-γ producing cells.

### CSFV neutralising and E2 specific antibodies detection

Serum samples taken at 0, 4, 8, 13, 15 and 21 dpv were tested by performing a neutralisation peroxidase-linked assay (NPLA) [23], and titres were expressed as the reciprocal dilution of serum that neutralised 100 TCID_50_ of the Catalonia strain in 50% of the culture replicates. The sera were also tested in the CSFV specific E2 ELISA (HerdChek CSFV Ab, IDEXX); when the blocking percentage ≥40%, the samples were considered positive, following the manufacturer’s recommendations.

### Detection of CSFV RNA

The RNA was extracted from all of the samples using the viral RNA isolation kit Nucleospin II according to the manufacturer's instructions (Macherey-Nagel). In all cases, an initial volume of 150 μL was used to obtain a final volume of 50 μL of RNA, which was stored at −80 °C. The presence of CSFV Catalonia strain RNA was analysed by RT-qPCR [24]. Positive results were considered for threshold cycle values (CT) equal or less than 42. Samples in which fluorescence was undetectable were considered negative. Furthermore, the presence of vaccine virus (C-strain) RNA was detected by RT-qPCR [7].

### ELISA for IFN-α detection in serum samples from the persistently infected-vaccinated group

To assess the innate immune response, serum IFN-α levels in the persistently infected-vaccinated pigs were evaluated in serum samples at 0, 4, 8, 13 and 21 dpv. Anti-IFN-α monoclonal antibodies (K9 and K17) and IFN-α recombinant protein (PBL Biomedical Laboratories, Piscataway, New Jersey, USA) were used in an ELISA assay to detect IFN-α in serum samples [22,25-27]. The cut-off value was calculated as the average optical density of negative controls (blank and negative serums before CSFV infection) plus three standard deviations. Cytokine concentrations in the serum were determined using a regression line built with the optical densities of the cytokine standards used in the test.

## Results

### Clinical signs after vaccination

**Figure 1:**
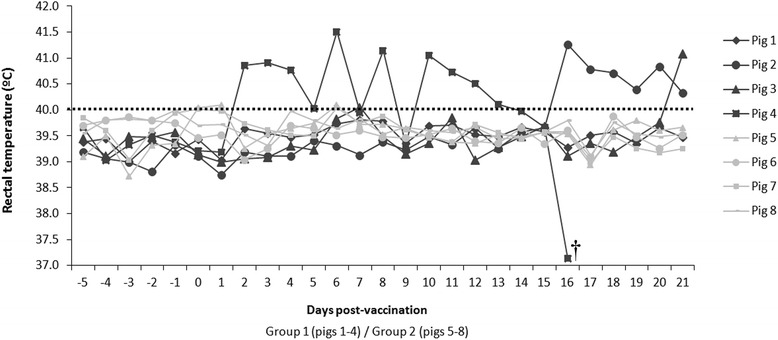
**Rectal temperature after vaccination.** The individual rectal temperature values were recorded daily after vaccination with a live attenuated vaccine (C-strain). Temperatures greater than 40 °C were considered fevers (indicated with a black dotted bar). CSFV persistently infected-vaccinated pigs (Group 1: numbers 1 to 4) and Pestivirus-Free vaccinated pigs (Group 2: numbers 5 to 8). † The pig was euthanized at 16 dpv.

**Figure 2:**
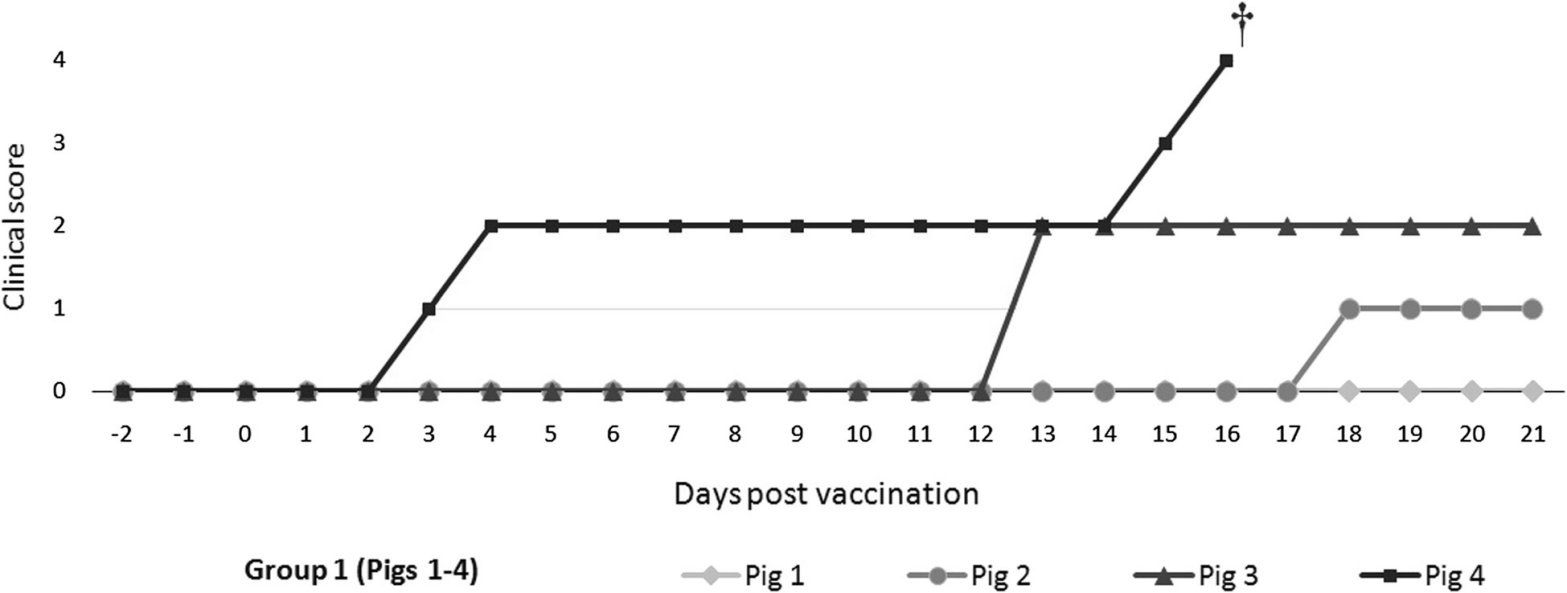
**Clinical score values in CSFV persistently infected-vaccinated pigs.** The individual clinical signs were recorded daily after vaccination until 21 days post-vaccination. The scores are defined in Section 2. † The pig was euthanized at 16 dpv.

After 21 dpv, no clinical signs were detected in vaccinated pigs from Group 2, and rectal temperatures remained within the established normal range until the end of the experiment, (Figure 1). Conversely, the vaccinated CSFV persistently infected pigs showed varying rectal temperature values; one of the pigs showed fever from day 2 until day 15 post-vaccination (pig #4), and had to be euthanized at 16 dpv after developing hypothermia, as well as severe clinical signs (diarrhoea, mild tremors, polyarthritis). Two pigs (#2 and #3) developed fevers starting at day 17 and 21 post-vaccination, respectively, in the absence of other clinical signs. Finally, pig #1 did not have an increase in rectal temperature at any point in the study with a healthy clinical status during the trial (Figures 1 and 2).

### Complete lack of response of the CSFV-specific IFN-γ producing cells from the persistently infected-vaccinated group

**Figure 3:**
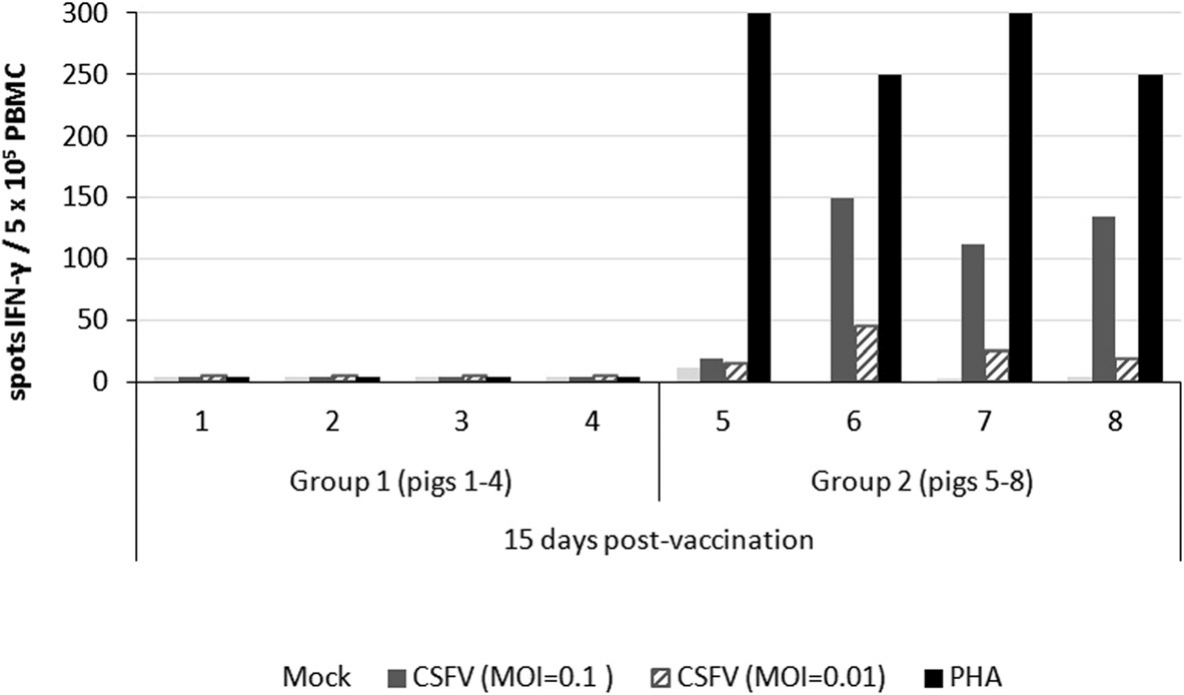
**CSFV-specific IFN-γ producing cells at 15 dpv.** CSFV persistently infected-vaccinated pigs (Group 1: numbers 1 to 4) and Pestivirus-Free vaccinated pigs (Group 2: numbers 5 to 8). Induction of CSFV-specific IFN-γ producing cells against different stimuli: mock, CSFV (MOI = 0.1 and MOI = 0.01, respectively) and PHA.

The ELISPOT assay results for the detection of IFN-γ in PBMC from persistently infected pigs (Group 1) showed a complete lack of response to stimulation against CSFV (MOI = 0.1 and 0.01) and PHA after 15 dpv (Figure 3). On the contrary, PBMC from vaccinated pigs in Group 2 showed a specific IFN-γ-producing cell response against CSFV and (PHA) stimuli (Figure 3).

### Absence of E2-specific antibodies and neutralising activity after vaccination of the persistently infected pigs

**Figure 4:**
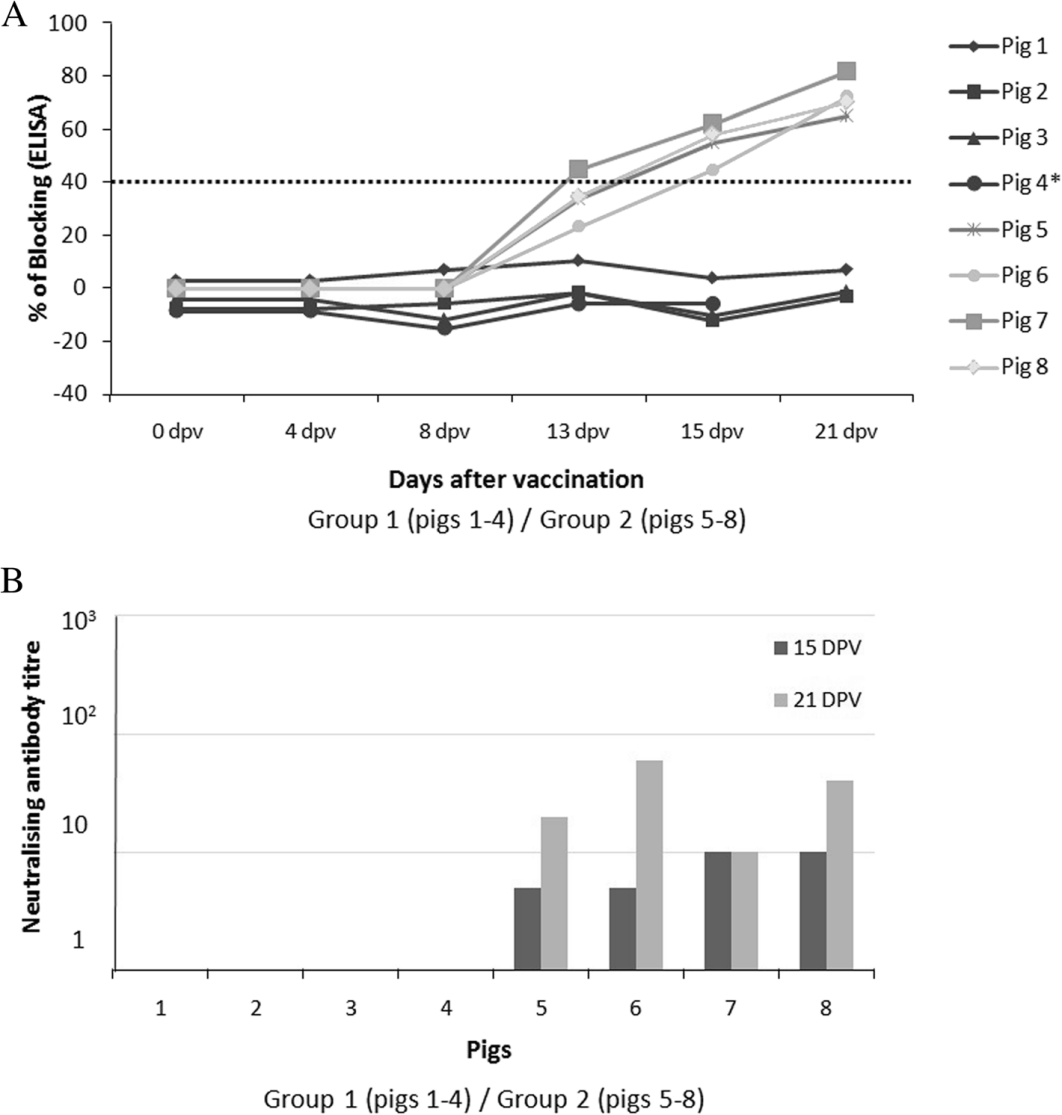
**E2-specific antibody detection and neutralising activity during 21 days post-vaccination.** CSFV persistently infected-vaccinated pigs (Group 1: numbers 1 to 4) and Pestivirus-Free vaccinated pigs (Group 2: numbers 5 to 8). **A**) Antibody response against the E2 glycoprotein detected by ELISA (in blocking %) at 0, 4, 8, 13, 15 and 21 days post-vaccination. Values greater than 40% were considered positive (indicated by a black dotted bar). **B**) Neutralising antibodies titres at 15 and 21 dpv. * This animal was euthanized at 16 dpv.

To evaluate the induction of CSFV-specific antibodies, serum samples were analysed at different times after vaccination. All vaccinated pigs from Group 2 showed E2-specific antibodies response detected by ELISA from 15 to 21 dpv (Figure 4A). Likewise, neutralising antibody titres were detected at 15 and 21 dpv (Figure 4B). In contrast, an absence of antibody response, in terms of E2-specific antibodies and neutralising titres, was found in all CSFV persistently infected-vaccinated pigs (Group 1) during the entire experiment (Figures 4A and B).

### CSFV RNA detection in serum, nasal and rectal swabs samples after vaccination

**Figure 5:**
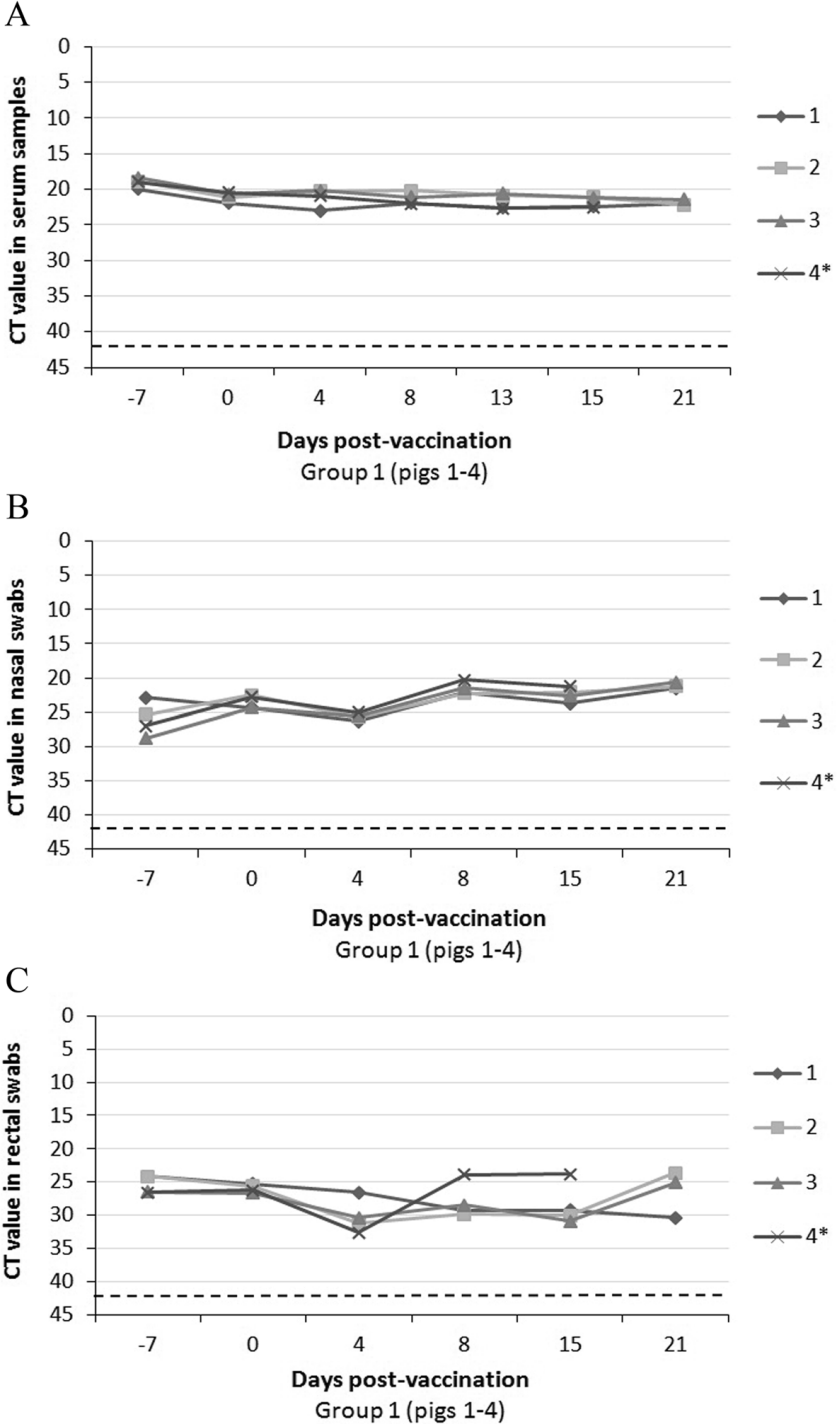
**Catalonia strain RNA detection in serum, nasal and rectal swabs at different times after vaccination with Hoffman assay.** CSFV persistently infected-vaccinated pigs (Group 1: numbers 1 to 4). **A**) Catalonia strain RNA detection in serum samples from CSFV persistently infected-vaccinated pigs at 0, 4, 8, 13, 15 and 21 dpv. **B**) Catalonia strain RNA detection in nasal swabs at 0, 4, 8, 15 and 21 dpv. **C**) Catalonia strain RNA detection in rectal swabs at 0, 4, 8, 15 and 21 dpv. A dotted bar indicates the detection limit of the technique above 42 CT. * This animal was euthanized at 16 dpv.

CSFV Catalonia strain-RNA was detectable in all of the samples analysed from postnatally persistently infected animals (Group 1) before vaccination until the end of the trial. A high level of Catalonia strain RNA was detected in serum samples throughout the study. Additionally, high amounts of Catalonia strain-RNA were detected in nasal and rectal excretions, as well as in the tonsils. There was a mostly constant amount of Catalonia strain RNA in serum samples and nasal swabs after one week post-vaccination (Figures 5A, B and C). In contrast, all vaccinated pigs from Group 2 were CSFV Catalonia strain-RNA negative throughout the experiment (data not shown).

### Lack of vaccine virus RNA detection in postnatally persistently infected pigs

CSFV vaccine virus RNA was detected in all of the sera samples analysed at 4 and 8 dpv and also in the tonsils from vaccinated pigs in Group 2. By contrast, a lack of vaccine virus (C-strain) RNA was detected in all of the samples tested from postnatally persistently infected-vaccinated pigs, including in the tonsils (Group 1) (Table 1).

**Table 1:** CSFV-vaccine RNA detection (C-strain) in serum at different times post-vaccination and in the tonsil samples

CT value in serum samples (Days post-vaccination)								CT value in tonsil samples^a^
Group of pigs	N° of pig	0 dpv	4 dpv	8 dpv	13 dpv	15 dpv	21 dpv	
Persistently infected-vaccinated pigs (Group 1)	1	Undetected	Undetected	Undetected	Undetected	Undetected	Undetected	Undetected
	2	Undetected	Undetected	Undetected	Undetected	Undetected	Undetected	Undetected
	3	Undetected	Undetected	Undetected	Undetected	Undetected	Undetected	Undetected
	4^b^	Undetected	Undetected	Undetected	Undetected	Undetected	-	Undetected
Pestivirus-Free vaccinated pigs (Group 2)	5	Undetected	39,34	39,68	40,41	Undetected	Undetected	26,27
	6	Undetected	40,10	40,20	40,05	Undetected	Undetected	26,07
	7	Undetected	39,11	39,12	Undetected	Undetected	Undetected	26,52
	8	Undetected	39,06	40,13	Undetected	Undetected	Undetected	28,32

^a^ Tonsil samples collected after the necropsy (21 dpv).

^b^ This animal was euthanized for ethical reasons at 16 dpv.

### Lack of IFN-α detection in serum samples from persistently infected-vaccinated group

In general, an absence of IFN-α was found in all of the serum samples analysed both before (day 0) and after vaccination from persistently infected-vaccinated pigs (data not shown). In the case of vaccinated pigs from Group 2, positive values were found only at 4 dpv ranged up to 40 U/mL (Data not shown).

## Discussion

It was shown over 40 years ago that congenital persistent infection is the most important cause by which CSFV is perpetuated in the domestic pig population [12]; however, little is known about the mechanisms involved. Currently, CSF causes significant losses in the pig farming industry worldwide, and despite the intensive control programs implemented in endemic countries for more than 20 years, the disease has not been eradicated in the world. Considering the complex epidemiology in endemic areas, viral evolution studies conducted in some of these zones that suggest the origin and circulation of low to moderate virulence strains, and the role that these types of strains may play as one important risk factor for the development of CSFV persistence in pigs [3,8,14,15,28-31], the existence of CSFV persistently infected pigs in endemic countries cannot be ruled out [16,17].

Recent work by our group demonstrated that CSFV persistently infected piglets can be generated by early postnatal infection either with a low or a moderate virulence CSFV strains [11].

Interestingly, the postnatally persistently infected animals became viraemic, excreting high viral loads during the six weeks of the study, but were unable to generate either humoral or cellular immune responses against CSFV. In the present work, we studied the immune response to a live attenuated vaccine (HCLV C-strain) in six-week-old CSFV persistently infected pigs, considering that this type of vaccine is routinely used in many of the endemic countries [3,7,8,32]. A complete lack of the vaccine viral RNA was detected in the serum samples and tonsils from CSFV postnatally persistently infected pigs during 21 days post-vaccination. Previous studies have shown that the tonsil is considered a target for the vaccine virus replication and wherein the vaccine virus persists for more than 30 days post-vaccination [4,33]. Furthermore, a lack of response to E2-specific antibodies and an absence of neutralising antibody titres were shown in CSFV persistently infected-vaccinated animals. Likewise, an absence of IFN-γ-producing cell response against CSFV or PHA was also observed. Considering the role played by IFN-γ in the control of CSFV infection [22,34], persistently infected-vaccinated animals maintained an immunosuppressive state. Herein lies one of the main differences between persistently and chronic infected pigs; in animals suffering from chronic infection, a CSFV-specific immune response is generated. Furthermore, the immunological anergy developed in postnatally persistently infected pigs supported the previously results described [11].

On the other hand, CSFV exacerbates the IFN-α response, which is detected in the serum of infected pigs; this response has been hypothesised to be related to disease severity rather than to protective immune responses [22,35]. Notwithstanding, IFN-α values were undetectable in the sera from postnatally persistently infected pigs after vaccination.

The absence of a CSFV-specific immune response generated following immunisation could be related to the apparent absence of replication of the vaccine virus in the samples analysed from these animals. Immunological tolerance has been described for CSF when the virus is transmitted in utero, leading to a persistently infected farrow [13]. Understandably, the pigs of this study (postnatally persistently infected), besides their immunological anergy [11], also might be immunotolerant. The blocking of a CSFV-specific immune response generated by the host should be beneficial for virus replication [36], as is the case for the CSFV Catalonia strain that induced the persistence in these animals. Paradoxically, the vaccine virus did not follow this logic, given the lack of viral detection in samples analysed after the vaccination of postnatally persistently infected pigs. Perhaps, the high viral load generated by the strain that induced the persistent infection (Catalonia strain) may be preventing the vaccine virus replication in the target tissues, changing or avoiding its replication capacity. Previous studies conducted in cell cultures with BVDV demonstrated that cells acutely infected with this virus were protected from the second infection by a homologous BVDV [36]. Probably, this interference phenomenon previously described in BVDV and CSFV in in vitro assays, would explain the lack of vaccine virus (C-strain) RNA detection in the samples analysed after the vaccination of postnatally persistently infected pigs, since the RNA from the vaccine strain could not enter into the host cells [37,38]. On the contrary and as expected, all vaccinated pigs in Group 2 were able to mount efficient humoral and cellular responses between 15 and 21 dpv [4,9,16,22,34], which can be associated with the RNA vaccine virus detection in the tonsil from these pigs. The efficacy of the C-strain vaccine in preventing clinical CSF seems to approach 100%. Most data from previous studies indicate a very high level of protection against the development of clinical signs after challenge, irrespective of the challenge strains used, even if the strains are from different CSFV genotypes [34,39-43]. Additionally, the C-strain vaccine (1.1 genotype) induced a detectable humoral response to CSFV [4,16].

The persistently infected-vaccinated pigs maintained high viral loads only for the CSFV Catalonia strain in serum throughout the study, and also had high amounts of viral RNA of this strain in nasal and rectal excretions. There was a constant amount of viral RNA in serum samples during the trial. The constant viraemia, as well as the high amount of viral excretion and the inability to induce a specific immune response, are common characteristics of *Pestivirus*-persistently infected animals [44]. However, contrary to CSFV postnatally persistently infected pigs, calves persistently infected with BVDV are able to develop immune responses against different pathogens, as well as respond to vaccination [45,46]. This fact suggests a different relationship between CSFV and its host despite both viruses being classified in the *Pestivirus* genus. The underlying factors for the development of clinical signs after a long period of incubation in persistently infected animals are unknown [47]. The late onset of disease has been described in this form, coursing with depression, anorexia, elevated temperature, conjunctivitis, dermatitis and locomotion disturbances [17]. Perhaps the vaccination could be a trigger for the disease progression, as would be the case for pig #4 (Figures 1 and 2). Before vaccination, this pig remained apparently healthy, but developed clinical signs (principally, fever peaks) two days post-vaccination (Figure 1). Our findings pose a better understanding of persistent infection with CSFV and also emphasise the need for diagnostic tools that can detect the existence of this CSF form in the field. Furthermore, our work supports once again that the vaccination strategies alone are not sufficient to eradicate the disease [4].

Considering their high levels of viral excretion, these animals can promote transmission to other healthy pigs in the herd, especially in situations where vaccination is not practiced or where the vaccination program is inefficient. Then, they can cause the short-cycle type of infection, which produces an acute fatal disease with high mortality [12]. Epidemiologically, it is not known how these pigs behave in the field, or the role they play in maintaining the infection in endemic countries, particularly important considering a population of 1% of persistently infected calves can maintain infection with BVDV in a farm [45]. There is still much more to know about CSFV postnatal persistent infection. Immunologically, we are only beginning to discover the mechanisms underlying the establishment of this form of disease; on a molecular level, it is known to be associated only with low-moderate virulence strains, but we still do not understand the reason why.

To our knowledge, this is the first report demonstrating the absence of a response to vaccination in pigs persistently infected with CSFV for 21 days post-vaccination. These results may have relevant implications for CSF control by vaccination. Likewise, these results might be of great value to understand the response to other persistent viral infections in humans and animals.

## References

[1] Dong XN, Chen YH (2007). Marker vaccine strategies and candidate CSFV marker vaccines. Vaccine.

[2] Moennig V, Floegel-Niesmann G, Greiser-Wilke I (2003). Clinical signs and epidemiology of classical swine fever: a review of new knowledge. Vet J.

[3] Pérez LJ, Díaz de Arce H, Perera CL, Rosell R, Frías MT, Percedo MI, Tarradas J, Dominguez P, Núñez JI, Ganges L (2012). Positive selection pressure on the B/C domains of the E2-gene of classical swine fever virus in endemic areas under C-strain vaccination. Infect Genet Evol.

[4] Ganges L, Núñez JI, Sobrino F, Borrego B, Fernández-Borges N, Frías-Lepoureau MT, Rodríguez F (2008). Recent advances in the development of recombinant vaccines against classical swine fever virus: cellular responses also play a role in protection. Vet J.

[5] Rümenapf T, Thiel HJ, Mettenleiter TC, Sobrino F (2008). Molecular biology of pestiviruses. Animal viruses: molecular biology.

[6] de Smit AJ, Bouma A, de Kluijver EP, Terpstra C, Moormann RJ (2000). Prevention of transplacental transmission of moderate-virulent classical swine fever virus after single or double vaccination with an E2 subunit vaccine. Vet Q.

[7] Liu L, Xia H, Everett H, Sosan O, Crooke H, Meindl-Böhmer A, Qiu HJ, Moennig V, Belák S, Widén F (2011). A generic real-time TaqMan assay for specific detection of lapinized Chinese vaccines against classical swine fever. J Virol Methods.

[8] Ji W, Niu DD, Si HL, Ding NZ, He CQ (2014). Vaccination influences the evolution of classical swine fever virus. Infect Genet Evol.

[9] Tarradas J, de la Torre ME, Rosell R, Perez LJ, Pujols J, Muñoz M, Muñoz I, Muñoz S, Abad X, Domingo M, Fraile L, Ganges L (2014). The impact of CSFV on the immune response to control infection. Virus Res.

[10] Allepuz A, Casals J, Pujols J, Jové R, Selga I, Porcar J, Domingo M (2007). Descriptive epidemiology of the outbreak of classical swine fever in Catalonia (Spain), 2001/02. Vet Rec.

[11] Muñoz-González S, Ruggli N, Rosell R, Pérez LJ, Frías-Leuporeau MT, Fraile L, Montoya M, Cordoba L, Domingo M, Ehrensperger F, Summerfield A, Ganges L (2015). Postnatal persistent infection of classical swine fever virus and its immunological implications. PLoS One.

[12] Liess B (1984). Persistent infection of hog cholera: a review. Prev Vet Med.

[13] van Oirschot JT (1977). A congenital persistent swine fever infection. II. Immune response to swine fever virus and unrelated antigens. Vet Microbiol.

[14] Díaz de Arce H, Ganges L, Barrera M, Naranjo D, Sobrino F, Núñez JI (2005). Origin and evolution of viruses causing classical swine fever in Cuba. Virus Res.

[15] Díaz de Arce H, Nuñez JI, Ganges L, Barreras M, Frías MT, Sobrino F (1998). An RT-PCR assay for the specific detection of classical swine fever virus in clinical samples. Vet Res.

[16] van Oirschot JT (2003). Vaccinology of classical swine fever: from lab to field. Vet Microbiol.

[17] van Oirschot JT, Terpstra C (1977). A congenital persistent swine fever infection. I. Clinical and virological observations. Vet Microbiol.

[18] Wensvoort G, Terpstra C, Boonstra J, Bloemraad M, Van Zaane D (1986). Production of monoclonal antibodies against swine fever virus and their use in laboratory diagnosis. Vet Microbiol.

[19] Reed LJ, Muench H (1938). A Simple method of estimating fifty per cent endpoints. Am J Epidemiol.

[20] Fan Y, Zhao Q, Zhao Y, Wang Q, Ning Y, Zhang Z (2008). **Complete genome sequence of attenuated low-temperature Thiverval strain of classical swine fever virus**. Virus Genes.

[21] Ganges L, Barrera M, Núñez JI, Blanco I, Frias MT, Rodríguez F, Sobrino F (2005). A DNA vaccine expressing the E2 protein of classical swine fever virus elicits T cells response that can prime for rapid antibody production and confer protection upon viral challenge. Vaccine.

[22] Tarradas J, Argilaguet JM, Rosell R, Nofrarías M, Crisci E, Córdoba L, Pérez-Martín E, Díaz I, Rodríguez F, Domingo M, Montoya M, Ganges L (2010). Interferon-gamma induction correlates with protection by DNA vaccine expressing E2 glycoprotein against classical swine fever virus infection in domestic pigs. Vet Microbiol.

[23] Terpstra C, Bloemraad M, Gielkens AL (1984). The neutralizing peroxidase-linked assay for detection of antibody against swine fever virus. Vet Microbiol.

[24] Hoffmann B, Beer M, Schelp C, Schirrmeier H, Depner K (2005). Validation of a real-time RT-PCR assay for sensitive and specific detection of classical swine fever. J Virol Methods.

[25] Diaz de Arce H, Artursson K, L’Haridon R, Perers A, La Bonnardiere C, Alm GV (1992). A sensitive immunoassay for porcine interferon-alpha. Vet Immunol Immunopathol.

[26] Nowacki W, Charley B (1993). Enrichment of coronavirus-induced interferon-producing blood leukocytes increases the interferon yield per cell: a study with pig leukocytes. Res Immunol.

[27] Guzylack-Piriou L, Balmelli C, McCullough KC, Summerfield A (2004). Type-A CpG oligonucleotides activate exclusively porcine natural interferon-producing cells to secrete interferon-alpha, tumour necrosis factor-alpha and interleukin-12. Immunology.

[28] Shen H, Pei J, Bai J, Zhao M, Ju C, Yi L, Kang Y, Zhang X, Chen L, Li Y, Wang J, Chen J (2011). Genetic diversity and positive selection analysis of classical swine fever virus isolates in south China. Virus Genes.

[29] Jiang DL, Gong WJ, Li RC, Liu GH, Hu YF, Ge M, Wang SQ, Yu XL, Tu C (2013). Phylogenetic analysis using E2 gene of classical swine fever virus reveals a new subgenotype in China. Infect Genet Evol.

[30] Sun SQ, Yin SH, Guo HC, Jin Y, Shang YJ, Liu XT (2013). Genetic typing of classical swine fever virus isolates from China. Transbound Emerg Dis.

[31] Moennig V (2000). Introduction to classical swine fever: virus, disease and control policy. Vet Microbiol.

[32] Huang YL, Pang VF, Lin CM, Tsai YC, Chia MY, Deng MC, Chang CY, Jeng CR (2011). Porcine circovirus type 2 (PCV2) infection decreases the efficacy of an attenuated classical swine fever virus (CSFV) vaccine. Vet Res.

[33] Kaden V, Lange E, Riebe R, Lange B (2004). Classical swine fever virus Strain ‘C’. How long is it detectable after oral vaccination?. J Vet Med B Infect Dis Vet Public Health.

[34] Graham SP, Haines FJ, Johns HL, Sosan O, La Rocca SA, Lamp B, Rümenapf T, Everett HE, Crooke HR (2012). Characterisation of vaccine-induced, broadly cross-reactive IFN-γ secreting T cell responses that correlate with rapid protection against classical swine fever virus. Vaccine.

[35] Summerfield A, Alves M, Ruggli N, de Bruin MGM, McCullough KC (2006). High IFN-alpha responses associated with depletion of lymphocytes and natural IFN-producing cells during classical swine fever. J Interferon Cytokine Res.

[36] Kane M, Golovkina T (2010). Common threads in persistent viral infections. J Virol.

[37] Lee YM, Tscherne DM, Yun SI, Frolov I, Rice CM (2005). Dual mechanisms of pestiviral superinfection exclusion at entry and RNA replication. J Virol.

[38] Mittelholzer C, Moser C, Tratschin JD, Hofmann MA (1998). Porcine cells persistently infected with classical swine fever virus protected from pestivirus-induced cytopathic effect. J Gen Virol.

[39] Aynaud JM, Launais M (1978). Hog cholera: immunization of young pigs with the Thiverval strain vaccine in the presence of colostral immunity. Dev Biol Stand.

[40] Vandeputte J, Too HL, Ng FK, Chen C, Chai KK, Liao GA (2001). Adsorption of colostral antibodies against classical swine fever, persistence of maternal antibodies, and effect on response to vaccination in baby pigs. Am J Vet Res.

[41] Kaden V, Renner C, Rothe A, Lange E, Hänel A, Gossger K (2003). Evaluation of the oral immunisation of wild boar against classical swine fever in Baden-Württemberg. Berl Munch Tierarztl Wochenschr.

[42] Suradhat S, Damrongwatanapokin S (2003). The influence of maternal immunity on the efficacy of a classical swine fever vaccine against classical swine fever virus, genogroup 2.2, infection. Vet Microbiol.

[43] Graham SP, Everett HE, Haines FJ, Johns HL, Sosan OA, Salguero FJ, Clifford DJ, Steinbach F, Drew TW, Crooke HR (2012). Challenge of pigs with classical swine fever viruses after C-strain vaccination reveals remarkably rapid protection and insights into early immunity. PLoS One.

[44] Peterhans E, Schweizer M (2010). Pestiviruses: how to outmaneuver your hosts. Vet Microbiol.

[45] Peterhans E, Schweizer M (2013). BVDV: A pestivirus inducing tolerance of the innate immune response. Biologicals.

[46] Bolin SR, McClurkin AW, Cutlip RC, Coria MF (1985). Response of cattle persistently infected with noncytopathic bovine viral diarrhea virus to vaccination for bovine viral diarrhea and to subsequent challenge exposure with cytopathic bovine viral diarrhea virus. Am J Vet Res.

[47] Liess B (Ed.) (1988) Classical swine fever and related Viral Infections. Series: Developments in Veterinary Virology, Vol. 5. Boston: Martinus Nijhoff Publishing

